# Supplementary material for: Leveraging insurance customer data to characterize socioeconomic indicators of Swiss municipalities
Source: PLoS One. 2021 Mar 3;16(3):e0246785. doi: 10.1371/journal.pone.0246785 (PMC7928527; doi:10.1371/journal.pone.0246785)
Supplement: S1 Appendix — (PDF) [file pone.0246785.s001.pdf]

Leveraging insurance customer data to characterize socioeconomic indicators of Swiss municipalities

Lorenzo Donadio[1], Rossano Schifanella[2, 3], Claudia R. Binder[1], Emanuele Massaro[1*,◕]

1 HERUS Lab, Ecole polytechnique federale de Lausanne, ENAC, IIE, GR C1 455 (Batiment GR) - Station 2, 1015 - Lausanne, Switzerland
2 University of Turin, Corso Svizzera 185, 10149, Turin, Italy
3 ISI Foundation, Via Chisola 5, 10126, Turin, Italy

* ema.massaro@gmail.com
◕Now at the European Commission Joint Research Centre, Ispra, Italy

Appendix

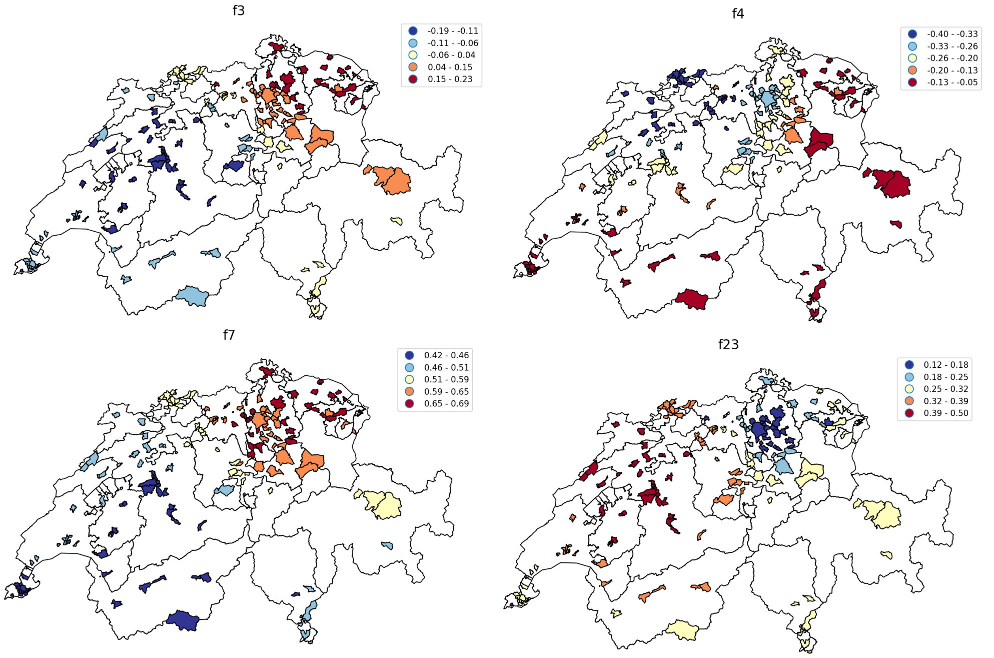

Fig A.1. GWR coefficients for all the features extracted as predictors for the variable **p1**.

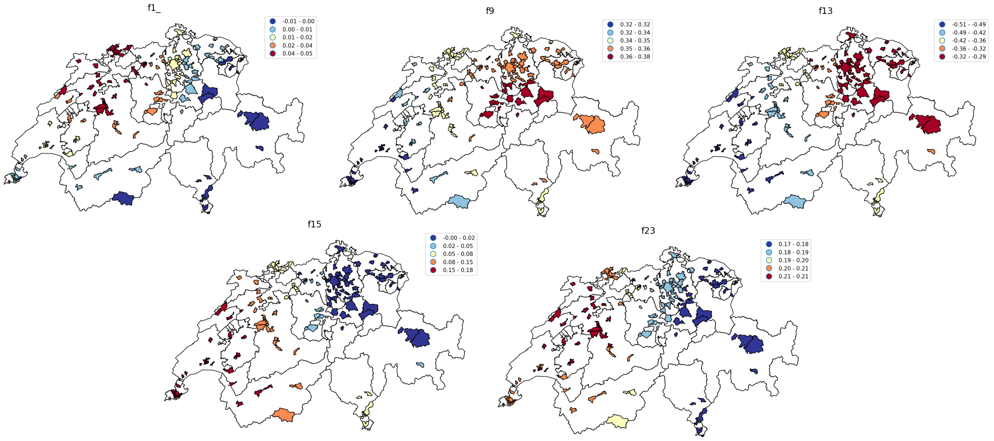

Fig A.2. GWR coefficients for all the features extracted as predictors for the variable **p2**.

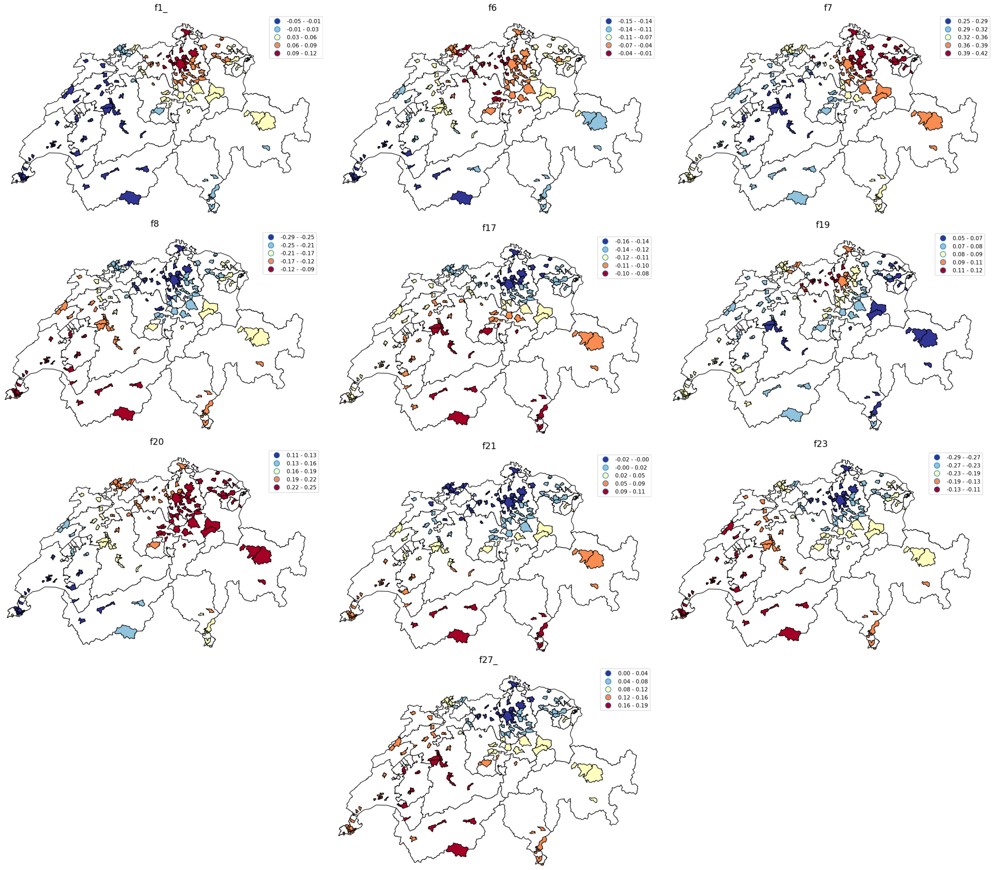

Fig A.3. GWR coefficients for all the features extracted as predictors for the variable **t1**.

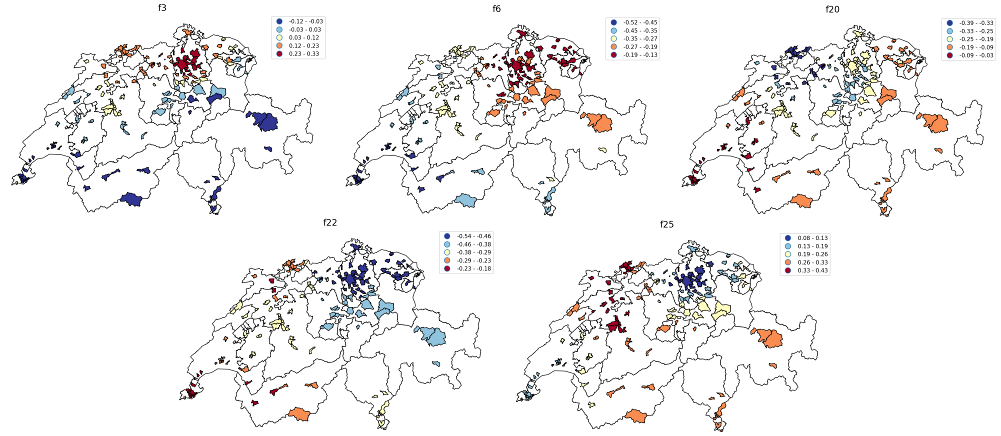

Fig A.4. GWR coefficients for all the features extracted as predictors for the variable **t2**.

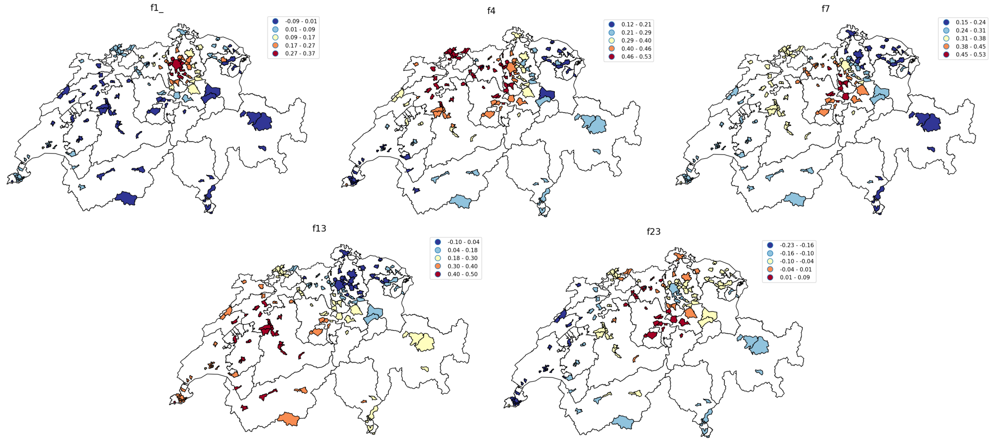

Fig A.5. GWR coefficients for all the features extracted as predictors for the variable **w1**.

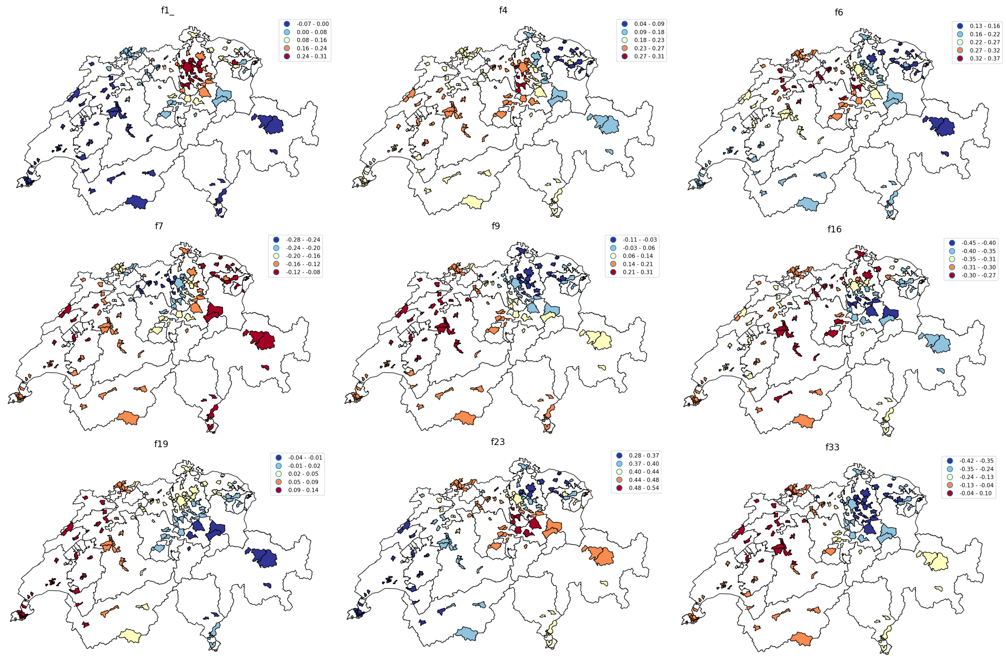

Fig A.6. GWR coefficients for all the features extracted as predictors for the variable **w2**.

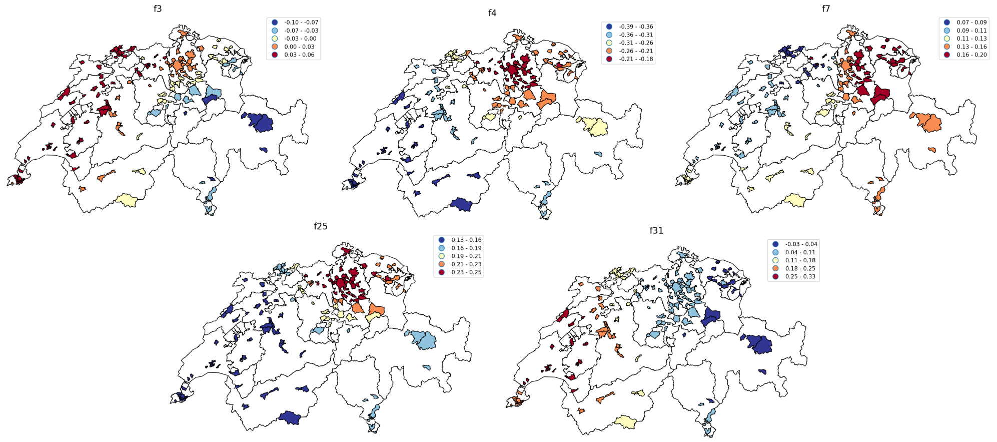

Fig A.7. GWR coefficients for all the features extracted as predictors for the variable **s1**.

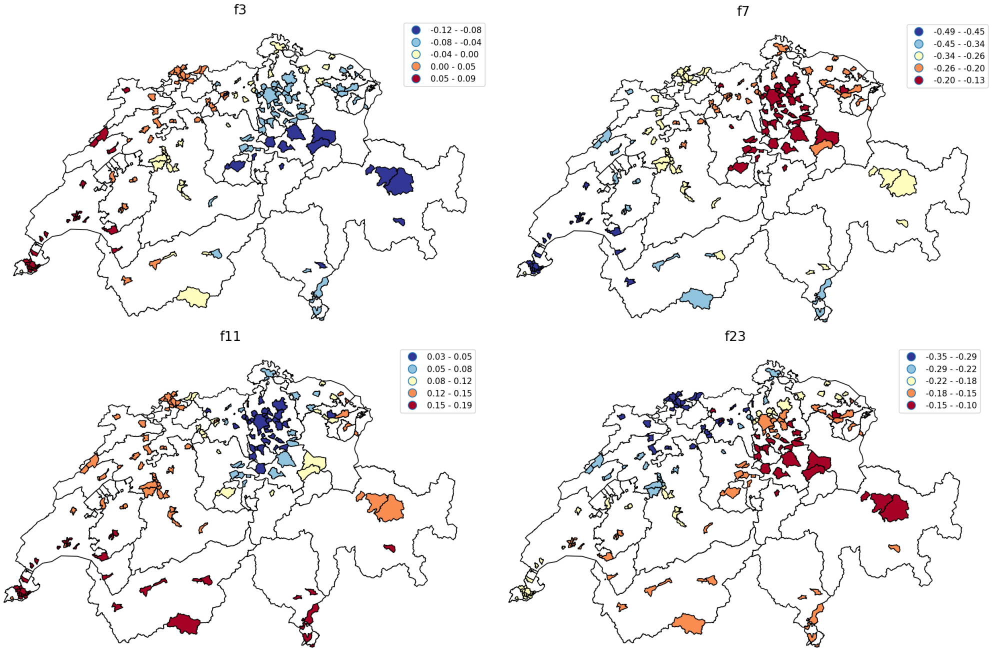

Fig A.8. GWR coefficients for all the features extracted as predictors for the variable **s2**.

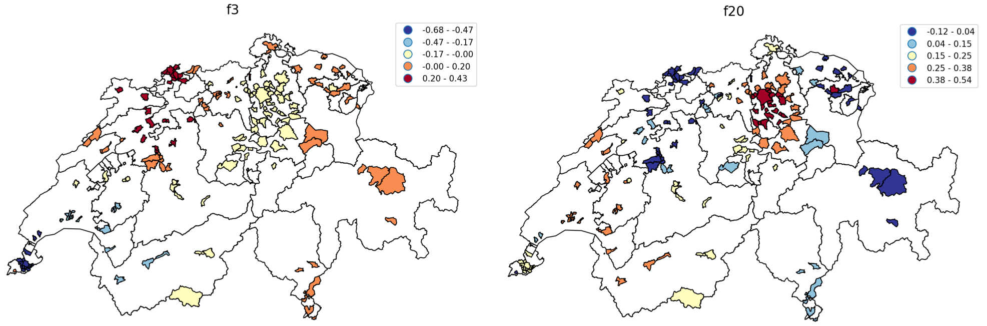

Fig A.9. GWR coefficients for all the features extracted as predictors for the variable **h1**.

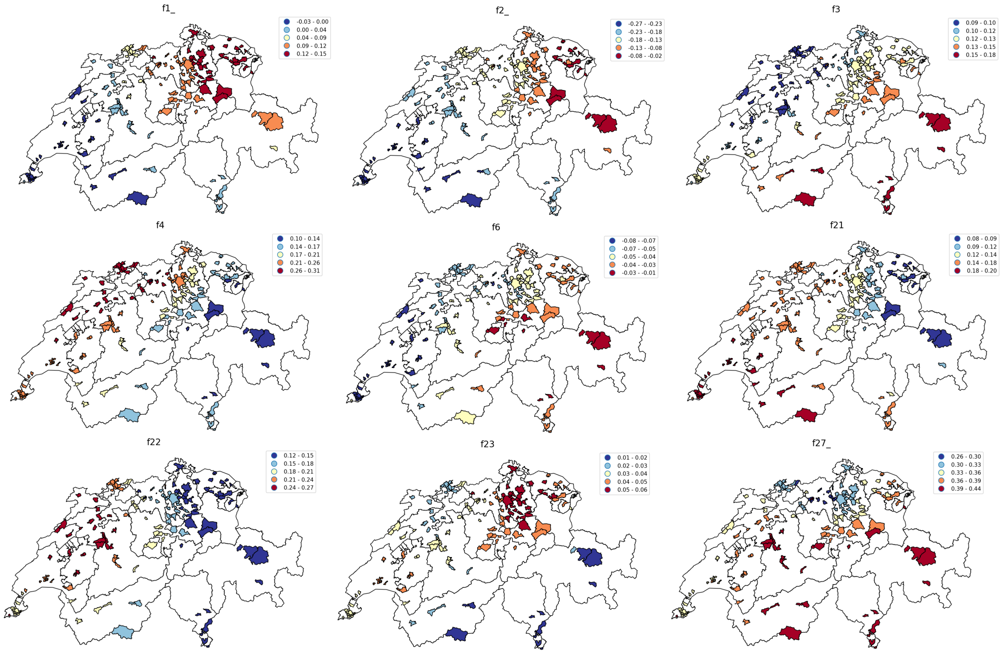

Fig A.10. GWR coefficients for all the features extracted as predictors for the variable **h2**.

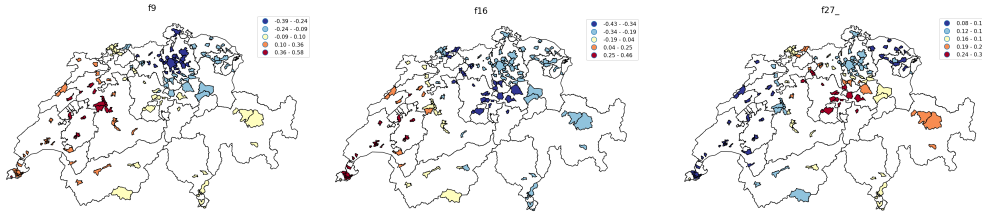

Fig A.11. GWR coefficients for all the features extracted as predictors for the variable **e1**.

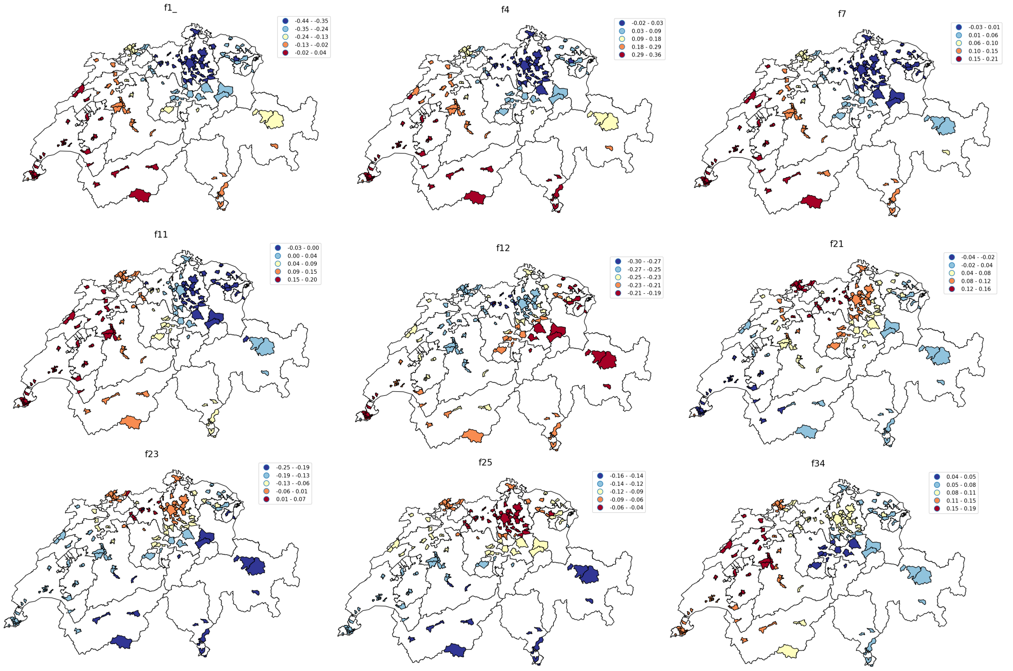

Fig A.12. GWR coefficients for all the features extracted as predictors for the variable **e2**.

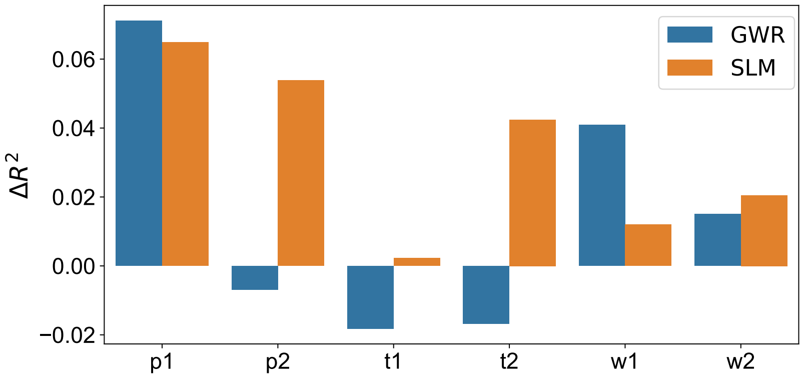

Fig A.13. Variation of the predictive performance due to the removal of not surprising features.
