# Supplementary material for: Leveraging insurance customer data to characterize socioeconomic indicators of Swiss municipalities
Source: PLoS One. 2021 Mar 3;16(3):e0246785. doi: 10.1371/journal.pone.0246785 (PMC7928527; doi:10.1371/journal.pone.0246785)
Supplement: S2 Appendix — (PDF) [file pone.0246785.s002.pdf]

Table A.1. Summary of the R^2 for the SLM model, using the Lasso selection algorithm. The average R^2 coefficient plus or minus its standard deviation is presented for the Training and Test sets.

	Full Dataset	Training Set	Test Set
p1	0.617	0.54 ± 0.0355	0.449 ± 0.0972
p2	0.639	0.623 ± 0.0179	0.542 ± 0.115
t1	0.493	0.49 ± 0.0736	0.157 ± 0.29
t2	0.727	0.53 ± 0.03	0.433 ± 0.117
w1	0.676	0.588 ± 0.00945	0.468 ± 0.0632
w2	0.712	0.65 ± 0.0139	0.543 ± 0.122
s1	0.412	0.365 ± 0.0514	0.278 ± 0.142
s2	0.447	0.369 ± 0.0395	0.14 ± 0.362
h1	0.312	0.0228 ± 0.0678	0.0432 ± 0.0608
h2	0.694	0.662 ± 0.545	0.0139 ± 0.136
e1	0.28	0.0927 ± 0.00859	0.0445 ± 0.128
e2	0.569	0.489 ± 0.224	0.0512 ± 0.24

Table A.2. Summary of the R^2 for the GWR model, using the Lasso selection algorithm. The average R^2 coefficient plus or minus its standard deviation is presented for the Training and Test sets.

	Full Dataset	Training Set	Test Set
p1	0.743	0.612 ± 0.0176	0.459 ± 0.108
p2	0.619	0.639 ± 0.0238	0.577 ± 0.112
t1	0.453	0.546 ± 0.0715	0.19 ± 0.188
t2	0.784	0.604 ± 0.0123	0.447 ± 0.156
w1	0.834	0.655 ± 0.019	0.42 ± 0.196
w2	0.818	0.707 ± 0.0199	0.463 ± 0.34
s1	0.454	0.421 ± 0.0642	0.237 ± 0.146
s2	0.616	0.429 ± 0.034	0.156 ± 0.243
h1	0.286	0.184 ± 0.0319	0.0561 ± 0.0964
h2	0.831	0.724 ± 0.0181	0.623 ± 0.152
e1	0.507	0.214 ± 0.0898	0.0326 ± 0.136
e2	0.649	0.623 ± 0.473	0.0332 ± 0.15

Table A.3. Summary of the coefficients for the Geographical Weighted Regression (GWR) for the variables p1, p2, t1, t2, w1 and w2

Variable	features	Mean	STD	Min	Median	Max
p1	Intercept	0.032	0.181	-0.415	0.037	0.343
Fraction of foreigners	f3	-0.180	0.188	-0.486	-0.214	0.459
	f4	0.556	0.114	0.115	0.575	0.693
	f7	0.286	0.165	-0.074	0.267	0.582
	f23	-0.040	0.269	-0.998	-0.035	0.419
p2	Intercept	0.020	0.018	-0.041	0.020	0.052
Fraction of	f1	0.347	0.022	0.296	0.353	0.398
beneficiaries of	f9	-0.410	0.140	-0.749	-0.364	-0.227
social assistance	f13	0.097	0.114	-0.058	0.060	0.369
	f15	0.192	0.021	0.135	0.192	0.226
	f23	-0.333	0.006	-0.340	-0.334	-0.313
t1	Intercept	0.015	0.064	-0.097	0.027	0.119
Cars per	f1	-0.088	0.037	-0.175	-0.071	-0.006
1000 inhabitants	f6	0.326	0.071	0.193	0.329	0.463
	f7	-0.169	0.080	-0.246	-0.212	0.008
	f8	-0.119	0.028	-0.167	-0.122	-0.009
	f17	0.072	0.015	0.006	0.075	0.094
	f19	0.181	0.082	-0.030	0.208	0.336
	f20	0.031	0.052	-0.020	0.012	0.264
	f21	-0.178	0.076	-0.282	-0.208	-0.038
	f23	0.120	0.062	0.004	0.108	0.260
	f27	0.162	0.018	0.123	0.164	0.210
t2	Intercept	0.005	0.313	-1.169	0.133	0.339
Fraction of commuters	f3	-0.306	0.156	-0.649	-0.248	0.007
using public	f6	-0.236	0.218	-0.896	-0.223	0.202
transportation	f20	-0.301	0.201	-0.586	-0.376	0.541
	f22	0.197	0.131	-0.019	0.175	0.580
	f25	0.274	0.115	-0.325	0.302	0.407
w1	Intercept	0.067	0.285	-1.716	0.069	0.557
Unemployment rate	f1	0.281	0.299	-1.688	0.388	0.547
	f4	0.302	0.127	-0.155	0.300	0.537
	f7	0.185	0.242	-0.571	0.099	0.917
	f13	-0.041	0.138	-0.687	-0.026	0.184
	f23	-0.213	0.260	-1.968	-0.229	0.247
w2	Intercept	0.052	0.137	-0.458	0.057	0.398
Unemployment rate	f1	0.190	0.110	-0.105	0.205	0.858
between women	f4	0.232	0.109	-0.355	0.233	0.371
	f6	-0.139	0.071	-0.298	-0.138	0.018
	f7	0.130	0.141	-0.129	0.116	0.398
	f9	-0.329	0.117	-0.944	-0.315	-0.133
	f16	0.046	0.097	-0.402	0.036	0.184
	f19	0.380	0.174	-0.012	0.412	1.186
	f23	-0.161	0.159	-0.402	-0.200	0.161
	f33	-0.300	0.151	-0.569	-0.318	0.078

Table A.4. Summary of the coefficients for the Geographical Weighted Regression (GWR) for the variables s1, s2, h1, h2, e1 and e2

Variable	features	Mean	STD	Min	Median	Max
s1	Intercept	0.027	0.114	-0.423	0.017	0.300
Building area (%)	f3	-0.303	0.090	-0.473	-0.266	-0.208
	f4	0.110	0.054	-0.014	0.107	0.228
	f7	0.161	0.067	0.019	0.179	0.248
	f25	0.202	0.219	-0.094	0.114	0.764
	f31	0.127	0.062	-0.062	0.155	0.184
s2	Intercept	-0.017	0.114	-0.490	-0.026	0.163
Green area (%)	f3	-0.307	0.165	-0.732	-0.235	-0.115
	f7	0.114	0.044	0.061	0.112	0.277
	f11	-0.196	0.103	-0.452	-0.182	0.104
	f23	-0.153	0.145	-0.319	-0.174	0.122
h1	Intercept	-0.004	0.510	-1.993	-0.047	1.293
Vacancy rate (%)	f3	0.106	0.350	-0.815	0.123	1.553
	f20	0.176	0.280	-0.781	0.083	0.757
h2	Intercept	0.060	0.092	-0.169	0.093	0.170
Average area	f1	-0.137	0.086	-0.290	-0.150	0.189
per inhabitant	f2	0.116	0.035	0.058	0.113	0.258
in square meters	f3	0.213	0.078	-0.003	0.219	0.377
	f4	-0.048	0.043	-0.134	-0.042	0.143
	f6	0.137	0.037	0.035	0.144	0.212
	f21	0.199	0.056	0.071	0.187	0.306
	f22	0.056	0.043	-0.040	0.048	0.197
	f23	0.361	0.074	0.180	0.361	0.615
	f27	-0.138	0.086	-0.305	-0.112	0.025
e1	Intercept	0.112	0.438	-0.859	-0.044	1.029
Municipal debt	f9	-0.021	0.383	-0.476	-0.246	0.893
	f16	0.153	0.181	-0.394	0.147	1.716
	f27	0.205	0.267	-1.335	0.174	0.793
e2	Intercept	-0.155	0.179	-0.360	-0.228	0.211
Fraction of investment	f1	0.161	0.126	0.013	0.105	0.422
in culture	f4	0.071	0.085	-0.035	0.055	0.263
	f7	0.089	0.111	-0.106	0.076	0.318
	f11	-0.216	0.054	-0.291	-0.228	-0.068
	f12	0.030	0.140	-0.366	0.080	0.174
	f21	-0.111	0.077	-0.332	-0.111	0.029
	f23	-0.081	0.030	-0.196	-0.078	-0.020
	f26	0.205	0.124	0.056	0.176	0.479
	f34	0.063	0.046	0.011	0.041	0.160
