# Supplementary material for: Leveraging insurance customer data to characterize socioeconomic indicators of Swiss municipalities
Source: PLoS One. 2021 Mar 3;16(3):e0246785. doi: 10.1371/journal.pone.0246785 (PMC7928527; doi:10.1371/journal.pone.0246785)
Supplement: Attachment — Submitted filename: PLOSONE - insurance - rebuttal.pdf [file pone.0246785.s003.pdf]

Leveraging insurance customer data to characterize socioeconomic indicators of Swiss municipalities

L. Donadio, R. Schifanella, C.R. Binder and E. Massaro

Rebuttal Letter

Comments from the Academic Editor Editor

Dear Dr. Massaro,

Thank you for submitting your manuscript to PLOS ONE. After careful consideration, we feel that it has merit but does not fully meet PLOS ONE's publication criteria as it currently stands. Therefore, we invite you to submit a revised version of the manuscript that addresses the points raised during the review process. Both qualified reviewers liked your paper and recommended a minor revision. Reviewer 1 mentioned the time dummy. I think you used only one-year data (2017). Reviewer 2 raised a few important technical and methodological issues. Please try to address their concerns as much as you can. I also have two minor expositional comments. (1) Some paragraphs are very long and it is not easy for readers. Please consider breaking a long paragraph into two or more paragraphs with each paragraph focusing on only one main point. (2) For tables 6 and 7, to ease readers' burden, it is better to add a column of variable names, for example, "fraction of owners" for f3. I feel it is troublesome to go back to table 4 to understand what f3 denotes. Also for each model, you can report the R-squared and the stability measure so that readers can have a better understanding of the model fitting.

Dear Editor,

Our thanks to you and the reviewers for your positive comments and constructive suggestions.

We have revised the manuscript addressing all the editorial comments. Please find the list of all the changes to the manuscript in our point-by-point response below. The original referees' comments are in bold. We share our findings and analysis with you and the reviewers in a public repository [https://github.com/emanuelemassaro/swissinsurance].

In this document, we tried to answer to all the comments made by reviewers by explaining the validity of our results and methodology.

Sincerely yours,

Lorenzo Donadio, Rossano Schifanella, Claudia R. Binder and Emanuele Massaro.

Response to reviewer 1

We thank the reviewer for appreciating our work and his interesting comments. In details:

1. The spatial distribution of insurance and Swiss Census data should be compared.

We thank the reviewer for this comment. The spatial distribution of the two datasets (insurance customers and Union of the Swiss Cities) is visually compared in Figure 1. We revised the caption in order to explain better the spatial differences between the two datasets.

2. Detailed methods in Features selection should be provided.

A description of the methodology adopted for the features selection step is reported in Section 4.1. We integrated the text with additional details on the alternative algorithms we tested and the motivations for the selection of the current pipeline. For space constraints, we provided references to the technical details of each features selection method adopted instead of explaining them from scratch in the manuscript.

3. In SLM and GWR model, time dummy should be added in.

Unfortunately, we have access to both the insurance and census data only for 2017 that makes it difficult to introduce any considerations on the temporal evolution of the phenomenon under study. The cross-sectional nature dataset doesn't easily allow to add a dummy time variable that would be more appropriate in the presence of different observations over time.

4. Why just use SLM model rather than SEM (Spatial error) model? Should provide examination result.

We thank the reviewer for this question that helps us to better explain an important point. Our goal was to compare a geographical regression model with local coefficients (GWR) against a spatial autoregressive model with global scope (SLM/SEM). In literature, different approaches have been adopted to find the most appropriate spatial econometric model specification given an empirical use case. In this work, following the discussion in [Pinkse10], we use a theoretical rather than a data-driven approach to guide the decision. In our scenario, we hypothesize the presence of global endogenous effects where the indirect influence of a spatial unit falls on the entire set of locations, producing high-order effects observable even in spatial units that are not directly connected. We adopt a lag model accordingly.

For completeness, we also run the Lagrange Multiplier (LM) [Anselin88] diagnostics based on the OLS residuals in their standard and robust forms that are at the core of the methodology described in [Anselin05]. Consistently with the hypothesis above, the selected specification was the SLM in all cases except for p1,p2, w1, and s1 where the LM was leading to the selection of

an SEM specification. To further explore the extent of this choice on performance, we compared the performance of the two alternative models across the set of target variables (see Figure 1).

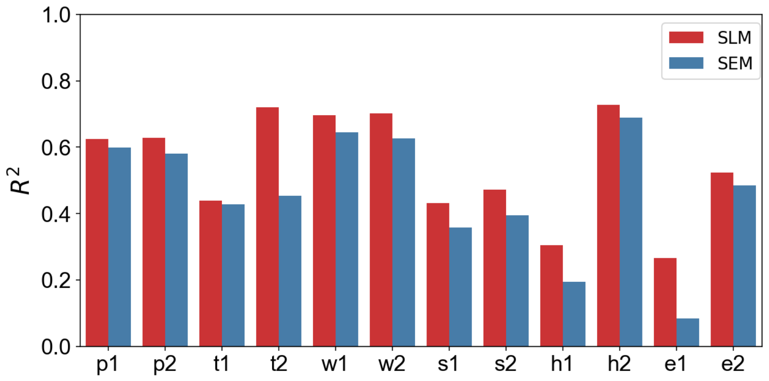

Figure 1

We observe that, consistently, the SLM specification leads to increased performance in the spatial regression task for all the targets in our scenario.

[Pinkse10] Pinkse, J., Slade, M.E.: The future of spatial econometrics*. Journal of Regional Science50 (1), 103–117(2010). doi:10.1111/j.1467-9787.2009.00645.x.https://onlinelibrary.wiley.com/doi/pdf/10.1111/j.1467-9787.2009.00645.x68.

[Anselin88] Anselin, L. Lagrange Multiplier Test Diagnostics for Spatial Dependence and Spatial Heterogeneity.Geographical Analysis (1988). doi:10.1111/j.1538-4632.1988.tb00159.x69.

[Anselin05] Anselin, L. Spatial Regression Analysis in R–A Workbook. Urbana (2005)

5. Why just use such spatial weight matrix as (5)? Can prepare the different results using different spatial weight matrices?

We thank the reviewer for this comment that allows us to explain in more detail the impact of the weighting scheme on the regression performance. First, it is worth noting that since the spatial units are not contiguous it is not possible to apply weighting schemes based on contiguity, e.g., Rook or Queen. Second, we focus on the adoption of different kernel functions that combine the distances between observations and calibration points placing more emphasis on observations that are closer than those farther away.

Due to space constraints, we were not able to add in the manuscript the results for each of the alternative weighting schemes, however, in Figure 2 we present the performance of the GWR model adopting three of the most widely used kernel functions: i.e, gaussian, exponential, and bi-square. In the results presented in the manuscript, we adopt an exponential kernel due to the highest or comparable to the highest performance achieved across the target variables.

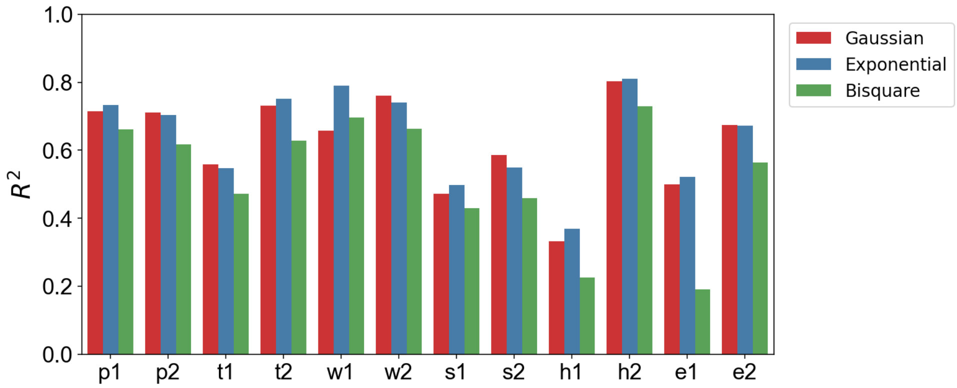

1.0
0.8
0.6
0.4
0.2
0.0
R^2
p1
p2
t1
t2
w1
w2
s1
s2
h1
h2
e1
e2
Gaussian
Exponential
Bisquare

Figure 2

Response to reviewer 2

On pages 4 and 5 in insurance data, the authors said that after matching the insurance data with the survey, 568,426 observations were left from the original dataset of 1,341,328 observations. Could the authors clarify why more than half observations were dropped when matching? Is that due to the lack of geographical identifiers in one of the data sources?

As described in Figure 1 and in Section 3, the mismatch between the spatial granularity and coverage of the two data sources is responsible for dropping part of the observations. In fact, the insurance customers data has a fine granularity at the level of zip code and high geographical coverage (La Mobiliere customers are spread across the country), while the official census data from the Statistics of Swiss cities contains information only on the municipalities with a population greater or equal to 10k. The 568,426 observations refer to the customers that live in one of those municipalities, leaving out the rest of the Swiss cities.

In accordance with a comment from Reviewer 1, we added a more detailed explanation of this phenomenon in the caption of Figure 1 in the manuscript.

On page 6 in Swiss census data. Could the authors give more information on the representativeness of the census data, for example, how many observations were collected in each municipality? Could the data be interpreted as representative at levels of municipalities or cantons or the nation?

As described in Section 3.2, the census data collected within the initiative Statistics of Swiss Cities that is the result of a collaboration between the Union of Swiss Cities and the Federal Statistical Office (FSO) contains multi-faceted statistics at the level of municipalities. The data comes from the official statistical office so it is considered representative and it is adopted in a wide range of studies and applications that involve the characterization of socioeconomic activities in Switzerland. We did not have access to detailed information on the procedure to compute each metric. You can find more information on the Swiss census corpus at the link https://uniondesvilles.ch/fr/bienvenue. The detailed description of the methods used by the Swiss Federal Statistical Office is available online[1]. All the information is in French or German. For instance if we consider the population data, it says:

---

[1] https://www.bfs.admin.ch/bfs/fr/home/statistiques/statistique-regions/portraits-regionaux-chiffres-cles/communes/donnees-explications.html

- *L'effectif de la population se réfère à la population résidante permanente à la fin de l'année. Source des données: Statistique de la population et des ménages STATPOP, Statistique du mouvement naturel de la population BEVNAT.*

  The size of the population refers to the permanent resident population at the end of the year. Data source: Statistics of population and households STATPOP, Statistics of natural movement of the population BEVNAT.

- ***Densité de la population par km2***
  Population mesurée par rapport à la surface des polygones (sans les lacs > 5 km2; swissBOUNDARIES3D © swisstopo).

  Population density per km2
  Population measured in relation to the polygon area (without lakes > 5 km2; swissBOUNDARIES3D © swisstopo).

On page 8 in feature selection. Could the authors explain the rationale using LASSO? Why not adaptive lasso or elastic net which may perform better than LASSO?

We thank the reviewer for this comment that is really relevant. Indeed, during our analysis, we analyzed different methods for features selection. We compared three alternatives: LASSO, ElasticNet, and Constrained LASSO. For all the models (OLS, GWR, and SLM) we compared the performance in terms of R2. We report the results in the figures below. We showed that consistently across models the Elastic Net features selection scheme achieves the highest performance, however, it requires a large number of features that makes the model difficult to interpret (16 dimensions). Moreover, in the case of the GWR model, we do not observe a significant difference in performance between LASSO and Elastic Net. Our approach in adopting the LASSO method across the experimental section was to strike a balance between performance, interpretability, and model complexity. The Constrained LASSO has been computed with the scikit-learn class `sklearn.linear_model.`**`MultiTaskLasso`**[2]

Feature Selection Methods	R2_mean (OLS)	R2_mean(GWR)	R2_mean(SLM)	# of features_mean
Lasso	0.48	0.64	0.54	5.9
Elastic Net	0.55	0.64	0.59	16.2
Constrained Lasso	0.47	0.56	0.57	23

[2] https://scikit-learn.org/stable/modules/generated/sklearn.linear_model.MultiTaskLasso.html

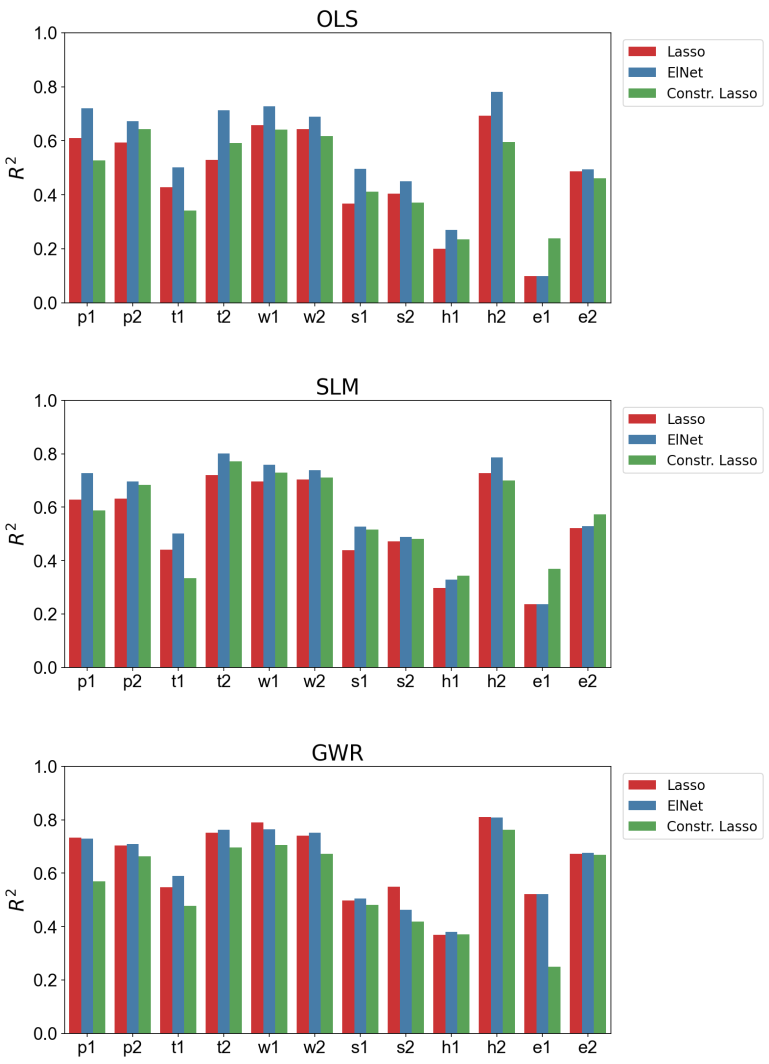

OLS
SLM
GWR
R^2
1.0
0.8
0.6
0.4
0.2
0.0
p1
p2
t1
t2
w1
w2
s1
s2
h1
h2
e1
e2
Lasso
ElNet
Constr. Lasso

Figure 3

The constrained Lasso method allows also to choose the number of selected features. In this response we show the results in terms of R2 for 31, 23, 16, 6 and 3 selected features for all the models.

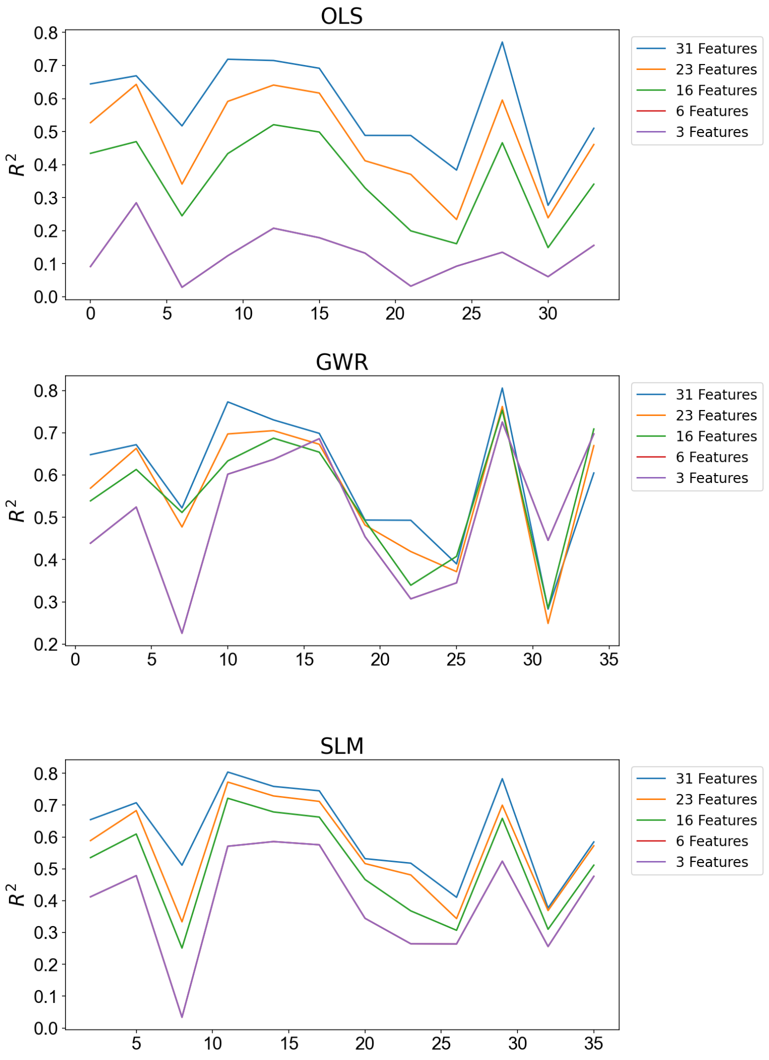

Figure 4

On page 9 in the spatial lag model. Please clarify the k-nearest neighbors (KNN) scheme? What is the weighting matrix regarding this scheme? Is it based on contiguity measures or kernels? Could the authors show the robustness of results concerning the alternative weighting scheme used?

Expanding the discussion on the robustness of the results to different weighting schemes started in a comment to Reviewer 1, we explore more in-depth the case of the Spatial Lag Model. In this context, we adopt a k-nearest neighbors (KNN) scheme and we optimize the hyperparameter *k* with a grid search approach that aims at finding the optimal value that

maximizes the average R2 across all the dependent variables. We use the PySAL package *spreg* to implement the SLM in our experimental settings. This method is based on a distance measure that creates the nearest neighbor weights matrix based on *k* nearest neighbors. Below we show the results for different schemes. First, we show the results for different values of *k* that do not influence the performance of the model for *k>1* as shown in the Figure below.

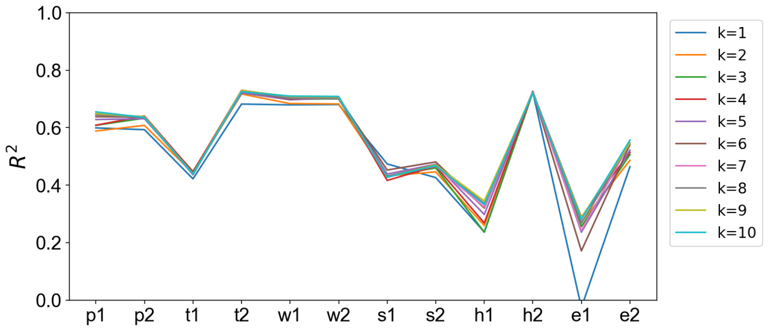

Figure 5

We also compare this scheme with an exponential kernel-based method and we show that there is no difference in terms of performance. As a reminder, we were not able to test the cases of weighting schemes based on contiguity measures, e.g., Rook and Queen, since the spatial units in our case are not contiguous.

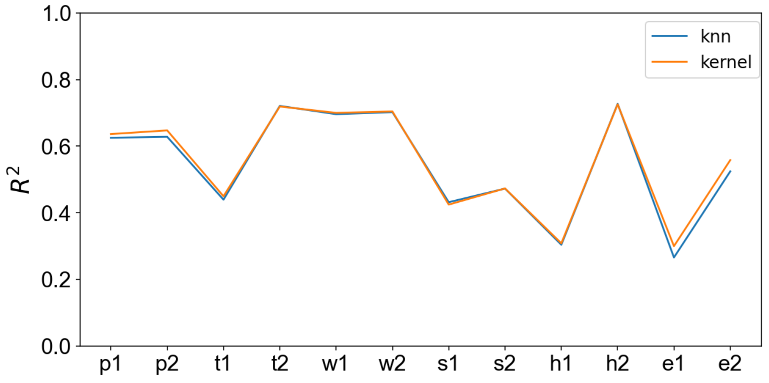

Figure 6

On page 10. Geographical Weighted Regression should be Geographically Weighted Regression.

We modified the manuscript accordingly.

On page 10. "In regression analysis, we try to explain the variations of a dependent variable using a suite of uncorrelated and normally distributed independent variables." This statement is not true. In most regression analyses, orthogonality and normality are not preconditions.

We removed from the manuscript the mention of the orthogonality and normality preconditions.

On page 9 in the methods. The predictive pipeline proposed by the authors consists of two steps. The first step is feature selection using LASSO, and the second is the prediction of census indicators using selected features. Although it's well-known that the OLS regression after LASSO (so-called OLS post-lasso estimator) performs very well in terms of its statistical properties \citep{Belloni2013}, the pipeline which uses more complicated models such as SLM or GWR after LASSO may behave differently. Could the authors provide references on the validity of this proposed pipeline?

We couldn't find a reference that adopted the exact same pipeline involving a step of features selection (LASSO) followed by a spatial regression model (SLM or GWR). We think that a theoretical proof or a detailed statistical validation is somehow outside the scope of the paper and not easy to integrate into the current manuscript. The rationale behind the implemented methodology was to coherently adopt the same features selection scheme independently of the regression model (OLS/SLM/GWR) in a way that the modeling step would have the same explanatory variables as input. In this direction, we treated the features selection process as a non-spatial task. In Section 4.1 and 5, we empirically measure the robustness and stability of this module in relation to the end-to-end regression performance. The main reason behind the introduction of the dimensionality reduction task is the presence of a high multicollinearity in case all the determinants in Table 3 are considered in the regression task. After the filtering, the impact of multicollinearity reduces substantially as well as the complexity of the models and, consequently, their interpretability.

On page 11 in the results. For each predictor, the selected features range from 2 to 9. It would be interesting to conduct a constrained LASSO selection where the number of features is constrained. Given that the aim of this paper is using insurance claim data as an alternative measure of census-based indicators, it would be more valuable if this could be achieved by a more sparse representation of selected features, say 2 or 3. I would appreciate it if the authors could check if this is the case.

We thank the reviewer for this relevant comment. To answer this question we compare three different features selection methods: 1) Lasso (that we used in the manuscript), 2) ElasticNet, and 3) Constrained Lasso. In doing so we used the or respective *scikit-learn* packages 1) LassoLarsIC[3], 2) ElasticNetCV[4] and 3) MultiTaskLasso[5]. As we can see in Figure 3 and Figure 4, a constrained Lasso method with 3 features is not able to perform well. Here we compare the performance of the constrained Lasso method with 3 features with the general Lasso procedure.

[3] https://scikit-learn.org/stable/modules/generated/sklearn.linear_model.LassoLarsIC.html
[4] https://scikit-learn.org/stable/modules/generated/sklearn.linear_model.ElasticNetCV.html
[5] https://scikit-learn.org/stable/modules/generated/sklearn.linear_model.MultiTaskLasso.html

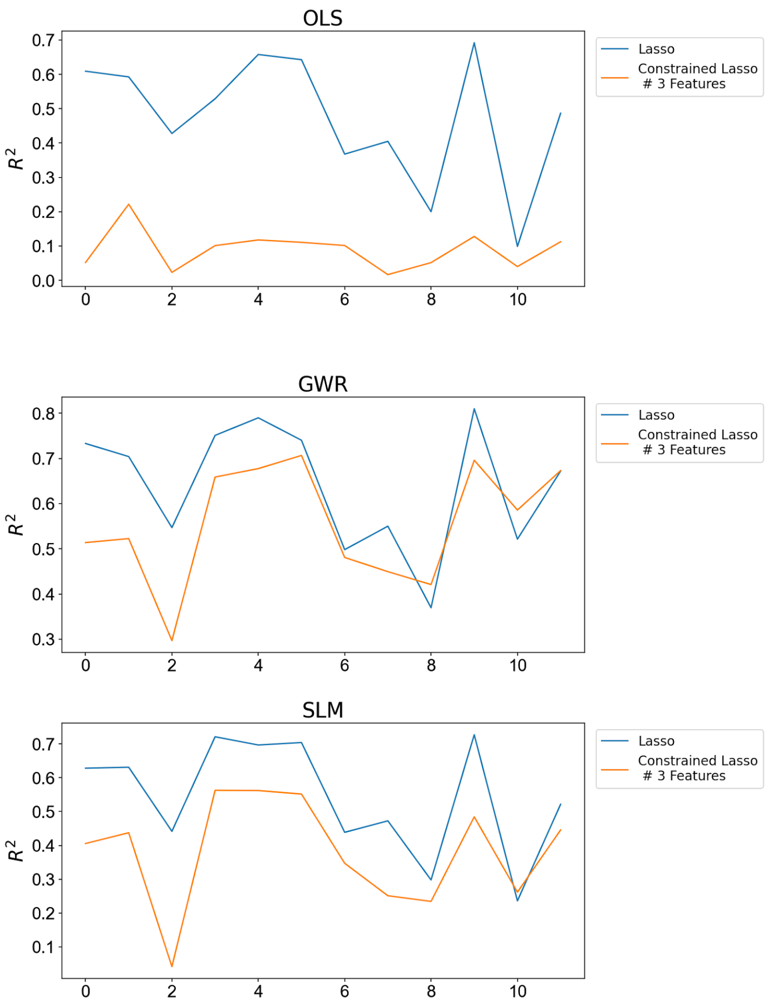

OLS
Lasso
Constrained Lasso
3 Features
R^2
GWR
Lasso
Constrained Lasso
3 Features
R^2
SLM
Lasso
Constrained Lasso
3 Features
R^2

Figure 7
